# *Centaurea triumfetii* essential oil chemical composition, comparative analysis, and antimicrobial activity of selected compounds

**DOI:** 10.1038/s41598-023-34058-2

**Published:** 2023-05-08

**Authors:** Ivana Carev, Andrea Gelemanović, Mateo Glumac, Klaudia Tutek, Mile Dželalija, Alessandro Paiardini, Gianni Prosseda

**Affiliations:** 1 Faculty of Chemistry and Technology, University of Split, Ruđera Boškovića 35, Split, Croatia; 2 NAOS Institute of Life Science, 355, Rue Pierre-Simon Laplace, 13290 Aix-en-Provence, France; 3 Mediterranean Institute for Life Science, Meštrovićevo šetalište 45, 2100 Split, Croatia; 4 School of Medicine, University of Split, Šoltanska 2, 21000 Split, Croatia; 5 Faculty of Sciences, University of Split, Ruđera Boškovića 33, Split, Croatia; 6 Department Biochemical Sciences “A. Rossi Fanelli”, University Sapienza, P.Le Aldo Moro 5, 00185 Rome, Italy; 7 Department of Biology and Biotechnology “Charles Darwin”, University Sapienza, Via Dei Sardi 70, 00185 Rome, Italy

**Keywords:** Secondary metabolism, Natural products

## Abstract

The essential oils from the *Centaurea* genus are well known for their pharmacological properties. The most abundant and dominant chemical components in *Centaurea* essential oils are *ß*-caryophyllene, hexadecanoic acid, spathulenol, pentacosane, caryophyllene oxide, and phytol. However, whether these dominant components are the key drivers for observed antimicrobial activity remains unclear. Thus, the aim of this study was dual. Here we provide comprehensive, literature-based data to correlate the chemical compounds in *Centaurea* essential oils with the tested antimicrobial activity. Secondly, we characterized the essential oil of *Centaurea triumfettii* All. squarrose knapweed using coupled system gas chromatography–mass spectrometry and tested its phytochemicals for antimicrobial activity against *E. coli* and *S. epidermis* using disc diffusion assay and monitoring their growth in Muller Hinton broth. The most abundant compounds in *C. triumfettii* essential oil were hexadecanoic acid (11.1%), spathulenol (10.8%), longifolene (8.8%), germacrene D (8.4%), aromadendrene oxide (6.0%) and linoleic acid (5.3%). Based on our analysis of literature data from other *Centaurea* essential oils, they were positively correlated with antimicrobial activity. Using an agar disk diffusion method, tested chemical constituents did not show experimental evidence to support this positive correlation to antimicrobial activity when we tested them as pure components. The antibacterial effect of essential oil constituents may be related to a complex synergistic, rather than a single component as suggested by performed network pharmacology analysis, underlying the theoretical interactions between the essential oil phytochemicals listed as potentially responsible for antimicrobial activity and should be confirmed in further in-depth studies. This is the first report on the comparative analysis of *Centaurea* essential oils with good antimicrobial activity, as well as the first analysis of chemical components of the essential oil from *C. triumfettii* and the first report of antimicrobial activity of the representative, pure components: aromadendrene, germacrene D, spathulenol, longifolene, and the mixture of selected chemical compounds. This work contributes to the body of knowledge on the genus *Centaurea* and *C. triumfettii* species.

## Introduction

Essential oils (EO) are complex mixtures of chemical components containing hundreds of chemical compounds. Although volatile terpenes, terpenoids, and hydrocarbons are usually the most representative EO molecules, their composition can significantly vary depending on plant species, growing conditions, and production procedures^1^. There has been a growing interest in essential oils and their components in recent years due to their potential biomedical or pharmaceutical applications^2,3^.

*Centaurea triumfettii* All*,* is commonly known as the Tocalote plant or squarrose knapweed. Studying *C. triumfettii* is essential for several reasons: ecological/biodiversity reasons, agricultural, and medicinal purposes. *Centaurea triumfettii* is an invasive species that spread from its native range in the Mediterranean region to other parts of the world. It competes with native vegetation for resources, reducing biodiversity and altering ecosystems. The plant can be a weed in agricultural fields and compete with crops for nutrients and space. Studying this plant can help identify effective control measures that reduce its impact on crop production. Studying biology, ecology, invasion biology and phytochemisty can help prevent the further spread of this species and control its population^4–7^.The plant’s ecological roles, such as its interactions with other plant species, pollinators, and herbivores, can contribute to our understanding of ecosystem dynamics. In communication with insects, essential oil plays an important role^2,6^. The Centaurea plant is used in traditional medicine for its medicinal properties, including antimicrobial, immunomodulatory, neuroprotective, antidiabetic antiviral, and anti-inflammatory biological activity^8,9^. *Centaurea triumfettii* phytochemistry and the biological activity of its extracts need to be better known. By studying the chemical composition of this plant, we can identify potential medicinal compounds that could be used in drug development.

Antimicrobial activity is one of the essential oils’ most investigated properties^10,11^. The genus *Centaurea* (family Asteraceae), comprising 400–700 different species distributed worldwide, is well known in ethnomedicine for its various pharmacological properties^12^. For this reason, EOs isolated from members of this family are promising candidates as a source of compounds with significant antimicrobial activity. Essential oils tested for antimicrobial activity, used for our analysis of literature data literature were isolated from *Centaurea affinis, C. aladagensis, C. alexandrina, C. amanicola, C. appendicigera, C. armena, C. behen, C. calcitrapa, C. cheirolopha, C. cineraria ssp. Umbrosa, C. consanguinea, C. damascena, C. glomerata, C. grisebachii ssp. Griebachii, C. haussknetchii, C. helenoides, C. jacea, C. lipii, C. lycopifolia, C. napifolia, C. nicaensis, C. pannonica, C. parlatoris, C. prosimopappa, C. pulcherrima var. pulcherrima, C. pullata, C. pumilio, C. ragusina, C. rupestris, C. scoparia, C. sessilis, C. solstitialis and C. tomentella*. This list comprises only 5–8% of the total known *Centaurea* species^13–32^. The antimicrobial activity of essential oils is often associated with their terpene compounds^33^. When an antimicrobial activity has been identified in these EOs, the dominant chemicals have been suspected to be responsible for this activity, even in the absence of clear evidence^9,11,33–38^. This assumption is somewhat speculative, as these studies usually do not test pure components dominant in essential oils, although the general understanding in pharmacology is that a particular chemical component can show significant biological activity, even at low concentrations, and that pure compounds could exhibit higher antimicrobial activity than plant extracts/essential oils^33,37,39^. However, we slowly begin to uncover that this might not be the case and that a particular chemical component can show significant biological activity, even at low concentrations^40^. Moreover, some studies look into the synergistic effect of a combination of compounds or among the plant extract/essential oil components which may explain the observed antimicrobial activity^41^.

Based on all available data on *Centaurea* essential oils’ antimicrobial activity, we conducted a correlation analysis between chemical constituents and antimicrobial activity to identify potential active chemical components. The main aim of this work was to verify whether the assumption that the main EO components, once isolated, are responsible for antibacterial activity is founded. To this end, we used both in silico and experimental approaches. On the other side, we defined the chemical composition of EO of *C. triumfettii*, squarrose knapweed, and once the main components were identified, we tested the antibacterial activity as pure chemical compounds. The obtained results are summarized below.

## Results and discussion

### *Centaurea* essential oil with antimicrobial activity: literature review

As a first step in our research, we reviewed the antimicrobial activity of 43 essential oils from 33 *Centaurea* species. The data were collected and extracted from the literature (the last literature search was performed in October 2022), and the database obtained can be found in Supplementary Table S1. With this literature review, we wanted to correlate which specific chemical compound is responsible for the antimicrobial activity of *Centaurea* essential oil.

We have identified a total of 417 different chemical compounds from 43 different essential oils analysed from the following species: *Centaurea affinis*, *C. aladagensis*, *C. alexandrina*, *C. amanicola*, *C. appendicigera*, *C. armena*, *C. behen*, *C. calcitrapa*, *C. cheirolopha*, *C. cineraria* ssp. *umbrosa*, *C. consanguinea*, *C. damascena*, *C. glomerata*, *C. grisebachii* ssp. *griebachii*, *C. haussknetchii*, *C. helenoides*, *C. jacea*, *C. lipii*, *C. lycopifolia*, *C. napifolia*, *C. nicaensis*, *C. pannonica*, *C. parlatoris*, *C. prosimopappa*, *C. pulcherrima* var. *pulcherrima*, *C. pullata*, *C. pumilio*, *C. ragusina*, *C. rupestris*, *C. scoparia*, *C. sessilis*, *C. solstitialis*, *C. tomentella.* These essential oils have been tested against a range of gram-positive and gram-negative bacteria, and some against fungi^13,14,16,20–22,25,27–32,42–47^.

We evaluated sorted data on antimicrobial activity using two approaches:

1. Correlating qualitative (presence or absence of chemical compounds) and quantitative (% of chemical compound) values of essential oils with antimicrobial activity, and.
2. Correlating only qualitative values (presence or absence of chemical compounds) of essential oils with antimicrobial activity.

**Figure 1:**
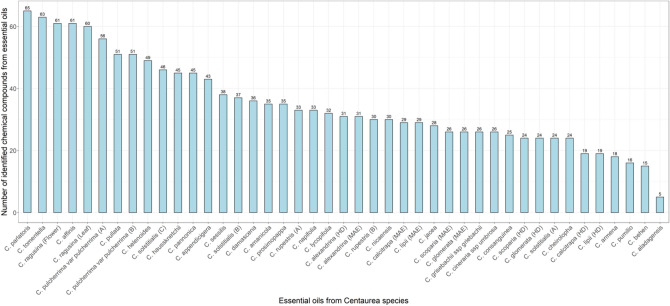
Number of chemical compounds identified in each studied essential oil from 33 *Centaurea* species. Data from the literature review.

After statistical processing, descriptive statistics showed that *C. parlatoris* had the highest number of identified chemical compounds—66; while *C. aladagensis* had the lowest number of identified chemical compounds—5 (Fig. 1).

**Figure 2:**
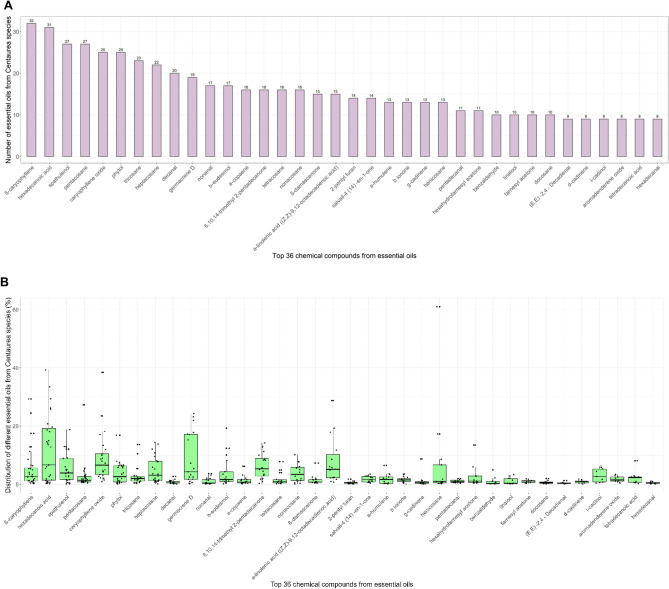
The chemical compounds commonly present in at least 9 out of 43 studied essential oil, from 33 different *Centaurea* species (**a**); and distribution of the amount (%, w/w) of commonly present chemical compounds in the studied essential oil (**b**). Data from the literature review.

Among the total of 417 chemical compounds detected, those found in the majority of *Centaurea* species were *ß*-caryophyllene (32/43 essential oils), hexadecanoic acid (31/43 essential oils), spathulenol (27/43 essential oils), pentacosane (27/43 essential oils), caryophyllene oxide (25/43 essential oils) and phytol (25/43 essential oils). The most common chemical compounds in *Centaurea* essential oils are shown in Fig. 2a.

The distribution of the commonly present chemical compounds in the studied essential oils showed high variability in the presence of hexadecenoic acid and germacrene D. In contrast, pentacosane and decanal showed low variability in their presence (Fig. 2b). The amount of each component is expressed as its frequency (%) in the essential oil. A complete summary of the number of identified chemical compounds in different *Centaurea* essential oils with their distribution is shown in Supplementary Table S2.

**Figure 3:**
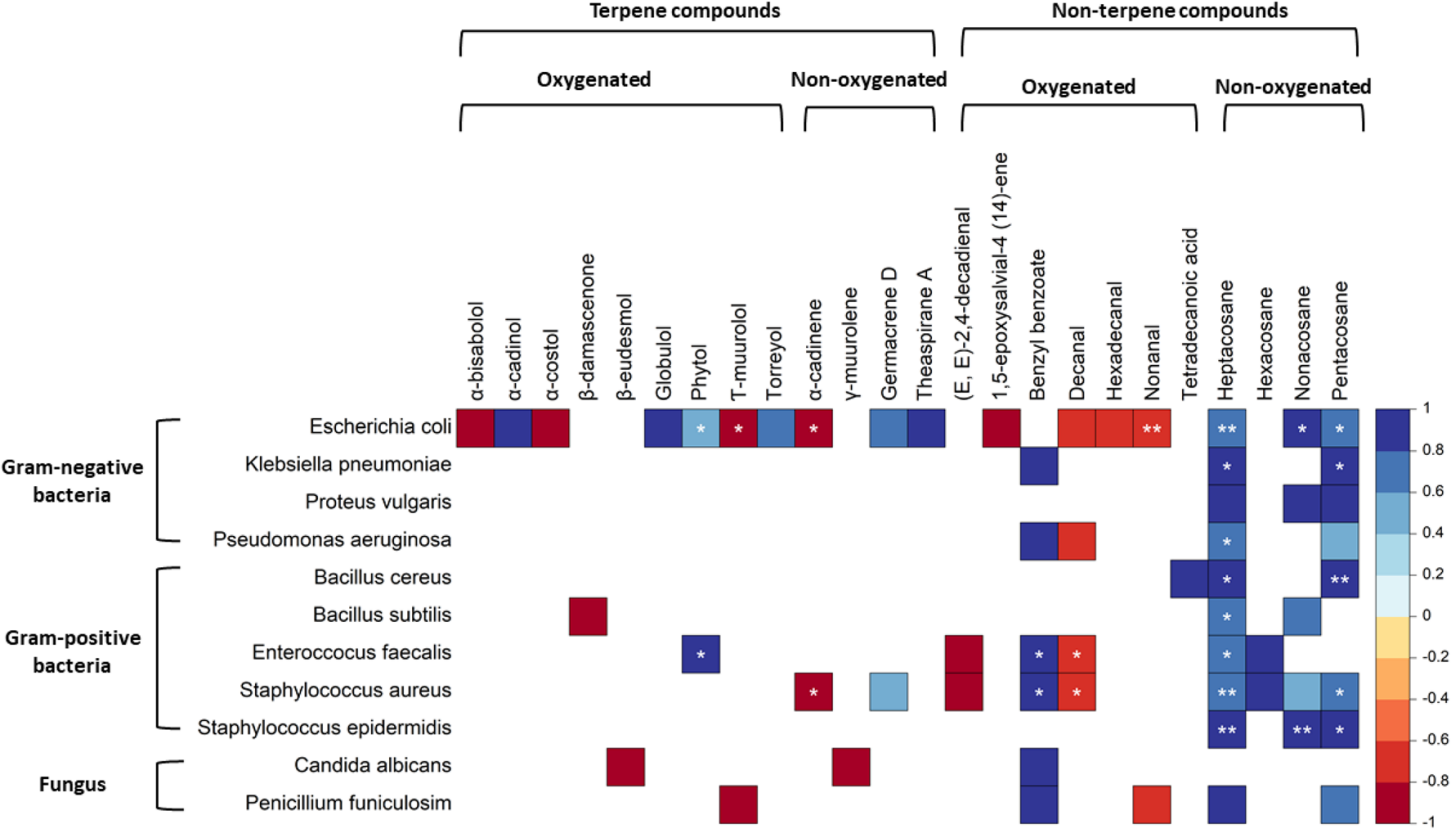
Correlating the qualitative (chemical compounds) and quantitative values of essential oils with good antimicrobial activity (MIC ≤ 500 µg/mL) of the essential oils (results depicted with Spearman’s rank correlation coefficient ranging from − 1, meaning strong negative correlation (red); to + 1 meaning strong positive correlation (blue); ** *p* < 0.01; * *p* < 0.05). Data from the literature review.

The next step in our statistical processing was to correlate the qualitative (chemical compounds) and quantitative (% or abundance of chemical compound) values of chemical compounds in *Centaurea* essential oils with good antimicrobial activity. This evaluation showed that the chemical compounds with a statistically significant positive correlation with antimicrobial activity were: heptacosane, pentacosane, nonacosane, benzyl benzoate, and phytol. (Fig. 3).

These compounds were found to have positive correlations with antimicrobial activity against 8 tested microbes: *E. coli, E. faecalis, S. aureus, S. epidermidis, B. cereus, P. aeruginosa, K. pneumoniae* and *B. subtilis*. Individual correlation scatterplots for statistically significant pairs (MIC value for specific microbes vs. abundance of specific chemical compound) can be found in Supplementary Fig. S1. Full correlation results are given in Supplementary Table S3.

When we extracted data for each microbe tested, we obtained different sets of chemical components that correlated with antimicrobial activity for each microbe, using both qualitative and quantitative values. Thus, the essential oils with the listed compounds: phytol, heptacosane, nonacosane, and pentacosane showed a positive correlation with antimicrobial activity, with good antimicrobial activity against *E. coli*. The same processing, in terms of good antimicrobial activity against *E. faecalis*, showed positive correlations with the antimicrobial activity value for phytol, benzyl benzoate and heptacosane; for *S. aureus*, showed positive correlations with the antimicrobial activity values for: benzyl benzoate, heptacosane, and pentacosane; for *S. epidermidis*, showed positive correlations with the antimicrobial activity values for *B. cereus* and *K. pneumoniae* showed positive correlations with antimicrobial activity values for heptacosane and pentacosane; while *P. aeruginosa* and *B. subtilis* showed positive correlations with antimicrobial activity values for: heptacosane. From the above, the heptacosane and pentacosane showed positive correlations with antimicrobial activity values for most of the microbes tested, namely *E. coli, K. pneumonia, B. cereus, S. aureus,* and *S. epidermidis* (Fig. 3).

Using only a qualitative approach, we have analyzed essential oils with antimicrobial activity, and the whole data set can be found in Supplementary Table S4. We decided to find a list of chemical compounds present in most essential oils (at least 30% of the data points) with good antimicrobial activity against a single microbe (Table 1). List of chemical compounds found in essential oils with good antimicrobial activity, using only qualitative approach, on the set of 7 tested microbes (*E. coli, S. aureus, P. aeruginosa, K. pneumoniae, E. faecalis, B. subtilis* and *C. albicans*) were: caryophyllene oxide, *ß*-caryophyllene, spathulenol, germacrene D, phytol, nonanal, decanal, hexadecanoic acid, heptacosane, nonacosane, pentacosane, tetracosane and tricosane (Table 1).

**Table 1:** Set of chemicals with potential antimicrobial activity on set of microbes analysed using only qualitative data.

Compound	Gram-negative bacteria			Gram-positive bacteria			Fungus	Number of microbes where specific compound showed good MIC (≤ 500 µg/mL)
	*E.coli* (N = 19)	*P. aeruginosa* N = 10)	*K. pneumoniae* (N = 9)	*S. aureus* (N = 26)	*E.faecalis* (N = 13)	*B. subtilis* (N = 11)	*C. albicans* (N = 10)	
	Presence of compound in essential oils that show good MIC (≤ 500 µg/mL)*							
Caryophyllene oxide	63.16%	60%	55.56%	50%	61.54%	81.82%	70%	7
Spathulenol	52.63%	60%	44.44%	65.38%	61.54%	72.73%	60%	7
*ß*-caryophyllene	63.16%	80%	88.89%	65.38%	84.62%	63.64%	80%	7
Nonanal	52.63%	70%	66.67%	38.46%	69.23%	36.36%	80%	7
Decanal	47.37%	60%	55.56%	34.62%	61.54%	45.45%	70%	7
Tricosane	42.11%	40%	33.33%	42.31%	46.15%	45.45%	50%	7
Tetracosane	31.58%	50%	44.44%	30.77%	46.15%	45.45%	50%	7
Pentacosane	57.89%	80%	66.67%	53.85%	76.92%	81.82%	80%	7
Heptacosane	47.37%	70%	55.56%	50%	69.23%	72.73%	70%	7
Nonacosane	31.58%	50%	33.33%	34.62%	46.15%	63.64%	40%	7
Hexadecanoic acid	68.42%	90%	77.78%	69.23%	69.23%	63.64%	80%	7
α-linolenic acid ((Z, Z)-9,12-octadecadienoc acid)	36.84%	60%	44.44%	30.77%	46.15%	45.45%	40%	7
α-copaene	36.84%	30%	33.33%	/	46.15%	54.55%	30%	6
β-ionone	31.58%	30%	/	30.77%	30.77%	54.55%	30%	6
β-eudesmol	31.58%	40%	/	30.77%	46.15%	45.45%	40%	6
Germacrene D	42.11%	90%	77.78%	38.46%	76.92%	/	70%	6
Phytol	52.63%	50%	55.56%	65.38%	53.85%	/	80%	6
ß-damascenone	36.84%	40%	/	38.46%	46.15%	45.45%	40%	6
Number of unique compounds present in essential oils that show good MIC	23	43	36	20	39	27	54	66 unique compounds

Data from the literature review (N = number of essential oils with good antimicrobial activity), results depicted for top 18 compounds found in at least 6/7 tested bacteria.

*Presence of chemical compound should be found in at least 30% of the studied essential oils with good antimicrobial activity.

The list of chemical compounds with potential antimicrobial activity against *E. coli*, present in at least 50% of the essential oils with good antimicrobial activity studied, is as follows: hexadecanoic acid, caryophyllene oxide, *ß*-caryophyllene, pentacosane, phytol, nonanal, and spathulenol.

The list of chemical compounds with potential antimicrobial activity against *P. aeruginosa* present in at least 60% of the essential oils with good antimicrobial activity studied is: hexadecanoic acid, germacrene D, *ß*-caryophyllene, pentacosane, nonanal, heptacosane, spathulenol, decanal, and caryophyllene oxide.

The list of chemical compounds with potential antimicrobial activity against *K. pneumoniae*, present in at least 60% of the essential oils studied with good antimicrobial activity, is as follows: *ß*-caryophyllene, hexadecanoic acid, germacrene D, pentacosane, and nonanal.

The list of chemical compounds with potential antimicrobial activity against *S. aureus* present in at least 50% of the essential oils with good antimicrobial activity studied is: hexadecanoic acid, *ß*-caryophyllene, spathulenol, phytol, pentacosane, heptacosane, and caryophyllene oxide.

The list of chemical compounds with potential antimicrobial activity against *E. faecalis*, present in at least 60% of the essential oils studied with good antimicrobial activity, is as follows: *ß*-caryophyllene, pentacosane, germacrene D, hexadecanoic acid, heptacosane, nonanal, decanal, spathulenol, and caryophyllene oxide.

The list of chemical compounds with potential antimicrobial activity against *B. subtilis* that were present in at least 50% of the essential oils studied with good antimicrobial activity is: caryophyllene oxide, pentacosane, heptacosane, spathulenol, *ß*-caryophyllene, hexadecanoic acid, and nonacosane.

The list of chemical compounds with potential antimicrobial activity against *C. albicans* that were present in at least 50% of the essential oils studied with good antimicrobial activity is: hexadecanoic acid, *ß*-caryophyllene, phytol, pentacosane, nonanal, caryophyllene oxide, decanal, heptacosane, germacrene D, and spathulenol.

**Figure 4:**
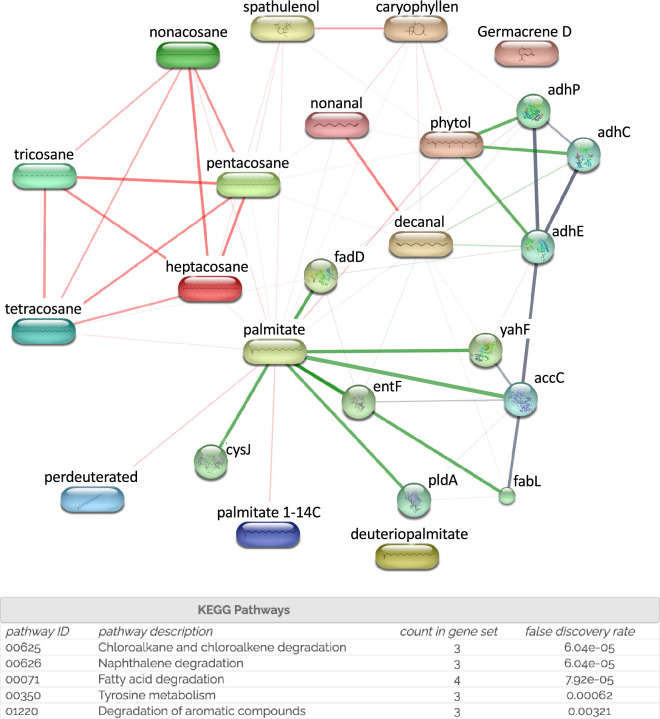
Network pharmacology analysis underlying the theoretical interactions between essential oil phytochemicals, and multiple bacterial target proteins, including fadD (acyl-CoA synthetase), cysJ (sulfite reductase), pldA (outer membrane phospholipase A), entF (enterobactin synthase multienzyme complex component), yahF (acyl-CoA synthetase with NAD(P)-binding domain), accC (acetyl-CoA carboxylase), fabl (enoyl-[acyl-carrier-protein] reductase), adhe (fused acetaldehyde-CoA dehydrogenase), adhC (alcohol dehydrogenase class III), adhP (ethanol-active dehydrogenase). The molecular targets were identified through the bioinformatics platform STITCH, which makes use of KEGG pathways (www.kegg.jp/kegg/kegg1.html). The components-targets plot was built through Cytoscape software.

As a final step in the evaluation of the literature data, we performed a network pharmacology analysis underlying the theoretical interactions between the essential oil phytochemicals listed as potentially responsible for antimicrobial activity, namely: hexadecanoic acid, phytol, caryophyllene, spathulenol, germacrene D, phytol, nonanal, decanal, heptacosane, nonacosane, pentacosane, tetracosane, tricosane, benzyl benzoate and microbial target proteins, which were comprised into the no single chemical database, a collection of manually drawn pathway maps representing our knowledge of the molecular interaction, reaction and relation networks (Fig. 4)^48,49^. The analysis revealed a complex network of interactions, mainly involving the degradation of fatty acids and aromatic compounds, as assessed by the KEGG pathways involved. This, in turn, suggests that the antibacterial effect of essential oil constituents may be partly related to a synergistic, rather than a single component, perturbation of the lipid fractions of (1) bacterial plasma membranes (this may affect membrane permeability and induce leakage of intracellular materials) and (2) fatty acids (essential sources of metabolic energy) metabolism.

After screening *Centaurea* essential oils for chemical compounds responsible for antimicrobial activity using both approaches, the differences in a set of chemical compounds with potentially good antimicrobial activity differ for each microbe, as well as the several single chemical compounds identified in at least 30% of essential oils with good antimicrobial activity (Table 1). Based on this criteria, 12 chemical compounds were found to be present in essential oils tested on 7 microbes (*E.coli, P. aeruginosa, K. pneumoniae, S. aureus, E. faecalis, B. subtilis, C. albicans*): caryophyllene oxide (median 61.5%; ranging from 50.0 to 81.8%), spathulenol (median 60.0%; ranging from 44.4 to 72.7%), *ß*-caryophyllene (median 80.0%; ranging from 63.2 to 88.9%), nonanal (median 66.7%; ranging from 36.4 to 80.0%), decanal (median 55.6%; ranging from 34.6 to 70.0%), tricosane (median 42.3%; ranging from 33.3 to 50.0%)**)**, tetracosane (median 45.5%; ranging from 30.8 to 50.0%), pentacosane (median 76.9%; ranging from 53.9 to 81.8%), heptacosane (median 69.2%; ranging from 47.4 to 72.7%), nonacosane (median 40.0%; ranging from 31.6 to 63.6%), hexadecanoic acid (median 69.2%; ranging from 63.6 to 90.0%), α-linolenic acid (median 44.4%; ranging from 30.8 to 60.0%). As we can observe, no single chemical compound is present in all essential oils with good antimicrobial activity.

We can conclude, based on our statistical analysis, that it is much more complicated to correlate the good antimicrobial activity of the essential oil to a specific chemical compound than just to link it to the most abundant chemical components of a specific essential oil, as it has been mentioned many times in the literature studied^13,14,16,20–22,25,27–32,42–47,50^.

Traditional relevant statistical methods for analysis of complex data sets include correlations between all pairs of data variables and finding principal components (i.e. PCA, the Principal Component Analysis), which in some cases can cause bias in the obtained^51^. This can happen because these methods are usually carried out by decomposing the covariance matrix of related variables, using only linear relationships between variables, valid only to a first-order approximation. For example, the bias can be caused by the normalization factors of different variables and by non-linear factors in correlations between variables. Therefore, further analysis would be required based on other types of correlation structures between variables.

The above list of chemical compounds in the composition of *Centaurea* essential oils that are correlated with a good antimicrobial activity can serve as a pharmacological guideline in future studies, and we cannot possibly say with certainty that these compounds are responsible for the antimicrobial activity until we examine their action in the form of pure compounds.

Of the prominent compounds that have been correlated with good antimicrobial activity using both approaches: caryophyllene oxide, *ß*-caryophyllene, spathulenol, germacrene D, phytol, nonanal, decanal, hexadecanoic acid, heptacosane, nonacosane, pentacosane, tetracosane, tricosane, and benzyl benzoate, only some has been tested on some microbes as a pure compounds: caryophyllene oxide, *ß*-caryophyllene, phytol, hexadecanoic acid, and benzyl benzoate^52–58^.

### *Centaurea triumfettii* essential oil chemical composition

The chemical composition of the essential oil of *C. triumfetti*i, expressed in terms of abundance (%) and determined by gas chromatography-mass spectrometry (GC/MS) coupled system, is presented in Table 2.

**Table 2:** Chemical compounds identified in *C. triumfettii* essential oil using GC/MS analysis.

Chemical compound	%, w/w	KI	Identification
Oxygenated monoterpenes			
Linalool	0.5	1090	KI, MS^59,60^
α-terpineol	0.6	1112	KI, MS^59,60^
γ-terpineol	0.7	1195	KI, MS^59,60^
Non oxygenated sesquiterpenes			
Bicycloelemene	0.9	1305	KI, MS^59,60^
* ß*-Ylangene	0.2	1351	KI, MS^59,60^
α-Copaene	0.3	1374	KI, MS^59,60^
Longifolene	8.8	1395	KI, MS^59,60^
Aromadendrene	1.4	1420	KI, MS^59,60^
* ß*-caryophyllene	2.3	1444	KI, MS^59,60^
Germacrene D	8.4	1467	KI, MS^59,60^
Pachulene	2.3	1490	KI, MS^59,60^
Oxygenated sesquiterpenes			
δ-cadinol	1.0	1540	KI, MS^59,60^
Spathulenol	10.8	1565	KI, MS^59,60^
Caryophyllene oxide	2.1	1589	KI, MS^59,60^
τ-cadinol	2.3	1642	KI, MS^59,60^
α-cadinol	4.5	1668	KI, MS^59,60^
Aromadendrene oxide	6.0	1694	KI, MS^59,60^
Oxygenated diterpene			
Phytol	2.9	2077	KI, MS^59,60^
Non terpene compounds			
Non oxygenated			
Tricosane	0.7	2300	KI, MS^59,60^
Tetracosane	0.5	2400	KI, MS^59,60^
Pentacosane	1.7	2500	KI, MS^59,60^
Hexacosane	1.8	2600	KI, MS^59,60^
Heptacosane	0.6	2700	KI, MS^59,60^
Octacosane	1.6	2800	KI, MS^59,60^
Nonacosane	0.3	2900	KI, MS^59,60^
Oxygenated compounds			
Aldehydes			
Benzaldehyde	4.9	953	KI, MS^59,60^
Benzacetaldehide	1.2	1048	KI, MS^59,60^
Acids			
Hexadecanoic acid	11.1	1983	KI, MS^59,60^
α-Linolenic acid	5.2	2143	KI, MS^59,60^
Oleic acid	1.6	2244	KI, MS^59,60^
Total	87.2		

*Kovac’s index (KI) determined on a VF-5MS column using the homologous series of n-alkanes (C9-C40), tr = traces (< 0.1%); MS = the compound was identified by MS; %, w/w (percent weight/weight), is relative quantitative ratio of chemical compound in the essential oil mixture, calculated from GC data, based on its peak area in a gas chromatogram.

A total of 30 components were identified in *C. triumfettii* essential oil, representing 87.2% of the total oil. The most abundant compounds were hexadecanoic acid (11.1%), spathulenol (10.8%), longifolene (8.8%), germacrene D (8.4%), aromadendrene oxide (6.0%) and linoleic acid (5.3%).

All of the dominant constituents found in *C. triumfettii* were also found as dominant constituents in *Centaurea* species, as shown by our statistical analysis of literature data^13,14,16,20–22,25,27–32,34–36,38,42–47,50^. All of the dominant components found in *C. triumfettii* have some correlation with antimicrobial activity, depending on the approach we used to evaluate the literature data statistically. We, therefore, decided to test the dominant components for antimicrobial activity as pure compounds, as none had been tested previously.

### Antimicrobial activity of *C. triumfettii* dominant chemical components

**Figure 5:**
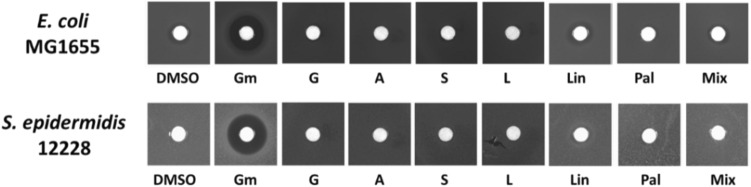
Agar diffusion test. The diffusion test was performed on Gram negative (strain *E. coli* MG1655) and Gram positive (*S. epidermidis* 12,228) bacteria. Paper disks were soaked with 5 µL of 10 mg/ml germacrene D (G), aromadendrene (A), spathulenol (S) and longifolene (L), and 2% linoleic acid (Lin) and palmitate (Pal). The mixed solution contains equivalent volumes of previously cited compound solutions (see Mat and Met). As for gentamicin, 5 µL of 1 mg/mL solution was used. Images are representatives of at least three independent experiments producing the same inhibition profile. Gm: Gentamicin, G: germacrene D, A: aromadendrene, S: spathulenol, L: longifolene, Lin: linoleic acid, Pal: palmitate (hexadecenoic acid), Mix: mix of the six compounds (G, A, S, L, Lin, Pal).

We have tested the dominant chemical compounds found in *C. triumfettii* for antimicrobial activity, using the agar diffusion method, against *E. coli* and *S. epidermis*, namely G: germacrene D, A: aromadendrene, S: spathulenol, L: longifolene, Lin: linoleic acid, Pal: palmitate (hexadecenoic acid) and a mixture of selected chemical compounds^61^. Antimicrobial activity, tested by the agar diffusion method, showed the absence of growth halos in all plates except the gentamicin control, indicating that none of the compounds tested, not even a combination of them, affected the survival of *E. coli* and *S. epidermidis* (Fig. 5).

**Figure 6:**
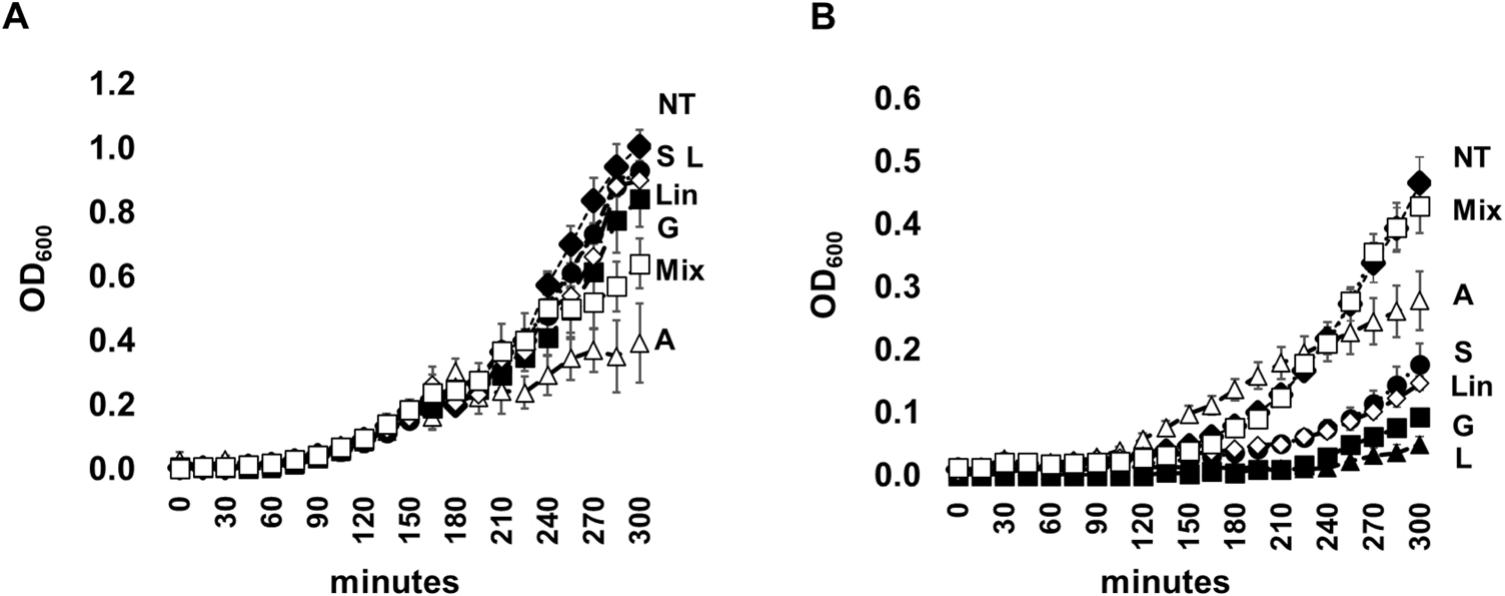
Monitoring of bacterial growth. Monitoring of bacterial growth of *E. coli* (A) and *S. epidermidis* (B) in the presence of 100 µg/ml aromadendrene (A), longifolene (L), germacrene D (G), and spathulenol (S), 0.02% linoleate (Lin) and a mixture (MIX) of all tested compounds. Bacterial monitoring was carried out for 300 min with readings every 15 min. Standard deviation bars are indicated. The data are the average of two independent experiments and each individual point was analysed in triplicate. A one-way ANOVA was performed to compare the effect of tested compounds on *E. coli* (*p* = 0.002) and *S. epidermidis* growth (*p* < 0.001).

The use of endpoint analyses such as the agar diffusion method runs the risk of underestimating the toxic effect of the compounds on the bacteria tested. To accurately assess the potentially toxic effects of the compounds studied, we decided to measure the influence that aromadendrene, germacrene D, spathulenol, longifolene, and linoleic acid could have on the bacterial fitness of *E. coli* MG1655 and S. epidermidis ATCC12228 strains by monitoring their growth in Muller Hinton broth for approximately 6 h. Unfortunately, it was impossible to analyse hexadecanoic acid as it was not soluble under the conditions and concentrations. The results of this analysis show that the bacterial growth of *E. coli* is partially inhibited by 100 µg/ml aromadendrene (Fig. 6A), whereas that of *S. epidermidis* is mainly sensitive to 100 µg/ml longifolene, spathulenol, germacrene D and 0.02% linoleic acid (Fig. 6B). However, despite the partial inhibitory effect on bacterial growth, the overall results show that the compounds tested do not exhibit sufficient toxicity to cause complete growth inhibition, even at the concentrations tested. Furthermore, the trend of the *E. coli* growth curves from 240 to 300 min, even in the presence of the two most affecting compounds (longifolene and germacrene D), suggests the increase of bacterial growth at successive time points.

More studies are investigating the antimicrobial properties of whole plant extracts and/or essential oils than pure compounds compounds^33^.Thus, in our study, we tested the dominant chemical compounds found in *C. triumfettii* as pure compounds, namely: germacrene D, aromadendrene, spathulenol, longifolene, linoleic acid, hexadecenoic acid, and a mixture of selected chemical compounds, however, none of these showed any significant inhibition of *E. coli* and *S. epidermis* growth. To our knowledge, these are the first results for these pure chemical compounds tested, except for hexadecenoic and linoleic acid^58,61^. Although some of the dominant components were very often correlated with the antimicrobial activity of the essential oil, and our statistical assessment of the literature data showed that some were strongly correlated with antimicrobial activity, we did not find experimental evidence for this statement.

Previous literature data have suggested that dominant compounds in essential oils cause antimicrobial activity. However, our research and correlation of dominant chemical components of essential oils in 33 *Centaurea* species showed that when using both quantitative and qualitative approaches, there was no significant correlation of antimicrobial activity with dominant compounds. On the contrary, there was a correlation when using only the qualitative approach. This confirms that the quantity of a particular chemical component is not the discriminating factor for the compound to be correlated with antimicrobial activity but rather its qualitative reference or presence. Some studies, like ours, showed that dominant components were not responsible for the antimicrobial activity of essential oil, such was the case with eucalyptus, thyme and lavender essential oils^62^.

In addition, this indication was confirmed in our study and disagrees with previous claims that dominant components are responsible for antimicrobial activity. Since *Centaurea* species contain characteristic dominant compounds in their essential oils, that would imply that all, or most, essential oils should have good antimicrobial activity. However, experimental data show that this is not the case. Furthermore, essential oils of *Centaurea* species with common dominant components in literature data showed to have different antimicrobial activity.

It would be necessary to identify common compounds of essential oils with good antimicrobial activity to narrow down the data set to the compounds with the highest chance of antimicrobial activity. As we can see, this task is not trivial, and the results vary depending on the method of literature data processing we use, as well as the sample size of the data. It is also essential to consider the way antimicrobial activity was tested, the microbial strains used, and the way of expression of antimicrobial activity.

## Conclusion

This is the first report on the comparative analysis of *Centaurea* essential oils with good antimicrobial activity, as well as the first analysis of chemical components of the essential oil from *C. triumfettii* and the first report of antimicrobial activity of the pure chemical components: aromadendrene, germacrene D, spathulenol, longifolene, and a mixture of mentioned chemical compounds with linoleic and hexadecanoic acids.

The first review, comparative analysis of *Centaurea* essential oils, and statistical correlation of the chemical composition and antimicrobial activity tested were done using a qualitative and quantitative approach. Using both approaches revealed 18 chemical components that could have potential antimicrobial activity. These compounds were: caryophyllene oxide, *ß*-caryophyllene, spathulenol, germacrene D, phytol, nonanal, decanal, hexadecanoic acid, heptacosane, nonacosane, pentacosane, tetracosane, tricosane and benzyl benzoate.

The first studied essential oil of *C. triumfettii* showed dominant chemical components: hexadecanoic acid (11.1%), spathulenol (10.8%), longifolene (8.8%), germacrene D (8.4%), aromadendrene oxide (6.0%) and linoleic acid (5.3%). These compounds were further tested on the antimicrobial activity as a pure compound and in the mixtures.

We have used the agar diffusion method to assess the potentially toxic effects of compounds on bacteria and a more comprehensive method to evaluate the toxic effect of compounds on bacteria, monitoring their growth in Muller Hinton broth for a few hours. However, the compounds tested did not exhibit sufficient toxicity to cause complete growth inhibition even at the concentrations tested.

Although some of the dominant components were very often correlated with the antimicrobial activity of the essential oil, and even in our statistical assessment, some were strongly correlated with antimicrobial activity, we did not find experimental evidence to support this statement.

As a result of our study, and observed in previous studies from literature data, we cannot associate antimicrobial activity with a single component. The antibacterial effect of essential oil constituents may be partly related to a synergistic, rather than a single component, perturbation of the lipid fractions of (1) bacterial plasma membranes (this may affect membrane permeability and induce leakage of intracellular materials) and (2) fatty acids (essential sources of metabolic energy) metabolism. This was suggested by performed network pharmacology analysis, underlying the theoretical interactions between the essential oil phytochemicals listed as potentially responsible for antimicrobial activity and should be confirmed in further in-depth studies.

The present data on the chemical composition of this species contributes to the body of knowledge on the genus *Centaurea*.

## Methods

### Plant material

*Centaurea triumfettii* All. plant material (leaves, stems, and flowers) were collected in July 2019. from wild growing populations in the Velebit mountain, Croatia (X = 44.70042617122714, Y = 14.983462013944544), at an elevation of 1622 m, growing on a dark brown soil type. The plant material authentication was carried out using macroscopic traits by botanist prof. Mirko Ruščić. Voucher specimens (2019_CTriumfettii_019) of plant materials used for this study have been deposited, with the date and location of collection, in the herbarium at the Department of Biochemistry, Faculty of Chemistry and Technology, Split, Croatia. On 4 February 2023, the plant name was checked with http://www.theplantlist.org for the last time.

### Extraction and chemical analysis of volatile oil

The volatile oil from air-dried aerial parts of plants was hydro-distilled using Clavenger apparatus for 3 h and stored in a sealed vial, under − 20 °C till use. The rate of distillation was 2.13% of essential oil and water to plant material ratio was 4:1.The gas chromatography analysis of VO was performed using Varian Inc. gas chromatograph, model 3900, Lake Forest, CA, USA equipped with a flame ionization detector and mass detector, model 2100 T, with a non-polar capillary column VF-5MS (30 m × 0.25 mm i.d.: coating thickness 0.25 m). Temperature program for VF-5MS column was: 60 °C isothermal for 3 min, then increased to 246 °C at a rate of 3 °C min-1 and held isothermal for 25 min. Carrier gas was helium at flow rate 1 mL min^−1^, injector temperature was 250 °C, injected volume 1 μL; split ratio of 1:20; FID detector temperature was 300 °C. Mass spectrometer ionization voltage was 70 eV, mass scan range: 40–350 mass units and ion source temperature were 200 °C. Identification of volatile oil chemical composition was based on comparison of their retention indices (relative to series of n-alkanes C9-C40), with internal database retention indices and literature retention indices using NIST2002 (National Institute of Standards and Technology, Gaithersburg, MD, USA) and on comparison of compound mass spectra with databases (Wiley 7 library—Wiley, New York, NY, USA)^59^. The internal database of compounds was created during previous analyses from authentic compounds obtained commercially and from more than thousand VO obtained during previous studies. The percentage of components was calculated as mean values from the GC and GC–MS peak areas.

### Bacterial strains and chemical compounds

Bacterial strains used in this study are *E. coli* MG1655, chosen as representative of gram-negative bacteria, and *S. epidermidis* ATCC12228, choosen as representative of gram-positive bacteria^63,64^. Linoleic and palmitic acid (BLD Pharmatech GmbH, purity > 94%), Germacrene D and Spathulenol (ChemFaces, purity >  = 98%), Aromadendrene oxide and Longifolene (Vitas-M Laboratory, Ltd., purity >  = 98%) were acquired by Molport Inc. All products were provided as a powder (5 mg, or 100 gr for palmitic acid) and dissolved in DMSO at a concentration of 10 mg/mL, except linoleic acid which was supplied as a liquid and diluted to 2% in DMSO.

### Antimicrobial testing

#### Agar diffusion method

Overnight bacterial cultures grown at 37 °C were diluted 100-fold in Muller Hinton broth and allowed to grow at OD600 of 0.1^64,65^. Then, 200 µl aliquots of these cultures were plated on Muller Hinton (MH) agar plates. 5 mm paper discs (Whatman 3MM Chr) containing 5 µL of 10 mg/ml germacrene D, aromadendrene, spathulenol and longifolene, 2% linoleic acid, and hexadecanoic acid were placed on the bacteria lawn and the plates were incubated overnight (16 h) at 37 °C. An additional plate was made using a mixture obtained by combining equivalent volumes of the tested compound solutions so having a 1.66 mg/mL final concentration for aromadendrene, germacrene D, spathulenol, and longifolene, 0.33% linoleic acid, and hexadecanoic acid. Two further plates were made for each bacterium: one with a paper disc containing only 5 µL of DMSO (the solvent used to dissolve all the compounds) and one with a paper disc containing 5 µL of gentamicin 1 mg/mL dissolved in water^66^.

### Monitoring the bacterial growth

Overnight bacterial cultures were diluted 1:100 in sterile MH broth with a final concentration of 100 µg/mL aromadendrene, germacrene D, spathulenol, and longifolene and 0.02% linoleic acid and hexadecanoic acid. In addition, we also considered a sample with only DMSO (not treated) and a sample with an iso-volumetric mixture of the tested compounds (1.66 ng/mL final concentration for aromadendrene, germacrene D, spathulenol, and longifolene, 0.0033% linoleic acid and hexadecanoic acid). Cultures of 100 µL were dispersed in a 96-well plate, each sample was analysed in triplicate. Growth curves were monitored for 3 h at 37 °C with automatic readings every 15 min via a multi-mode plate reader (CLARIOstar^®^ Plus, MGB).

### Statistical analysis

The literature data were collected from Scopus and PubMed databases, using keywords: “*Centaurea*”, “essential oil”, “antimicrobial activity”, with last literature screening performed on 30 October 2022.

All statistical analyses were performed using the free software environment for statistical computing R version 4.0.0^67^. Basic descriptive statistics was performed to examine the distribution and composition of examined essential oils from various *Centaurea* plant species. To test the association between the composition of essential oils and the antimicrobial activity of *Centaurea* plant species, we applied two approaches:

#### Qualitive and quantitative approach

We extracted all essential oils from *Centaurea* plant species for which there were available information of their antimicrobial activity expressed as minimal inhibitory concentration (MIC). The MIC number is the lowest concentration (in μg/mL) of an essential oil that inhibits the growth of a given strain of bacteria. Spearman’s rank correlation was applied to find correlations between MIC values and presence and amount of specific chemical compound identified from *Centaurea* essential oils for all compounds identified from each *Centaurea* plant species. Only statistically significant correlations (defined with *P* value < 0.05) or potentially suggestive correlations (defined with *P* value < 0.1), and those for which there was minimum of 4 data points available were discussed later. We performed this approach on all microbes for which there was at least 4 data points available.

#### Qualitive approach

With this approach we have assessed only essential oils with good antimicrobial activity and presence of specific chemical compound identified from *Centaurea* essential oils with good antimicrobial activity. If essential oil displayed MIC values less than or equal to 500 μg/mL, the antimicrobial activity was considered as good; while above 500 μg/mL the antimicrobial activity was considered weak or low. Data were presented with numbers and percentages to check which compounds (regardless of their proportion in the essential oil) are more abundantly present in essential oils that show good antimicrobial activity. We also tried to apply Fisher’s exact test to test if there is any statistical significance between the two newly formed categorical variables, however, we were very limited with the amount of data that show low antimicrobial activity. We performed this approach only on microbes for which we had at least 10 data points.

Pharmacology Network Analysis (PNA) was carried out using STITCH software, whereas the components-targets plot was built using the software Cytoscape version 3.6.

### Plant material collection

Plant material, aerial parts of *C. triumfetii* were collected in Croatian natural habitats according to Croatian National Legislation, for scientific research, following the rule of not damaging whole population, which was taken into special consideration when collecting plant material.

## Supplementary Information

Supplementary Information 1.

Supplementary Information 2.

Supplementary Information 3.

Supplementary Information 4.

Supplementary Information 5.

## Data Availability

The datasets used and/or analyzed during the current study available from the corresponding author on reasonable request.
