# Supplementary material for: Centaurea triumfetii essential oil chemical composition, comparative analysis, and antimicrobial activity of selected compounds
Source: Sci Rep. 2023 May 8;13:7475. doi: 10.1038/s41598-023-34058-2 (PMC10167351; doi:10.1038/s41598-023-34058-2)
Supplement: Supplementary file 1 — Supplementary Information 1. [file 41598_2023_34058_MOESM1_ESM.pdf]

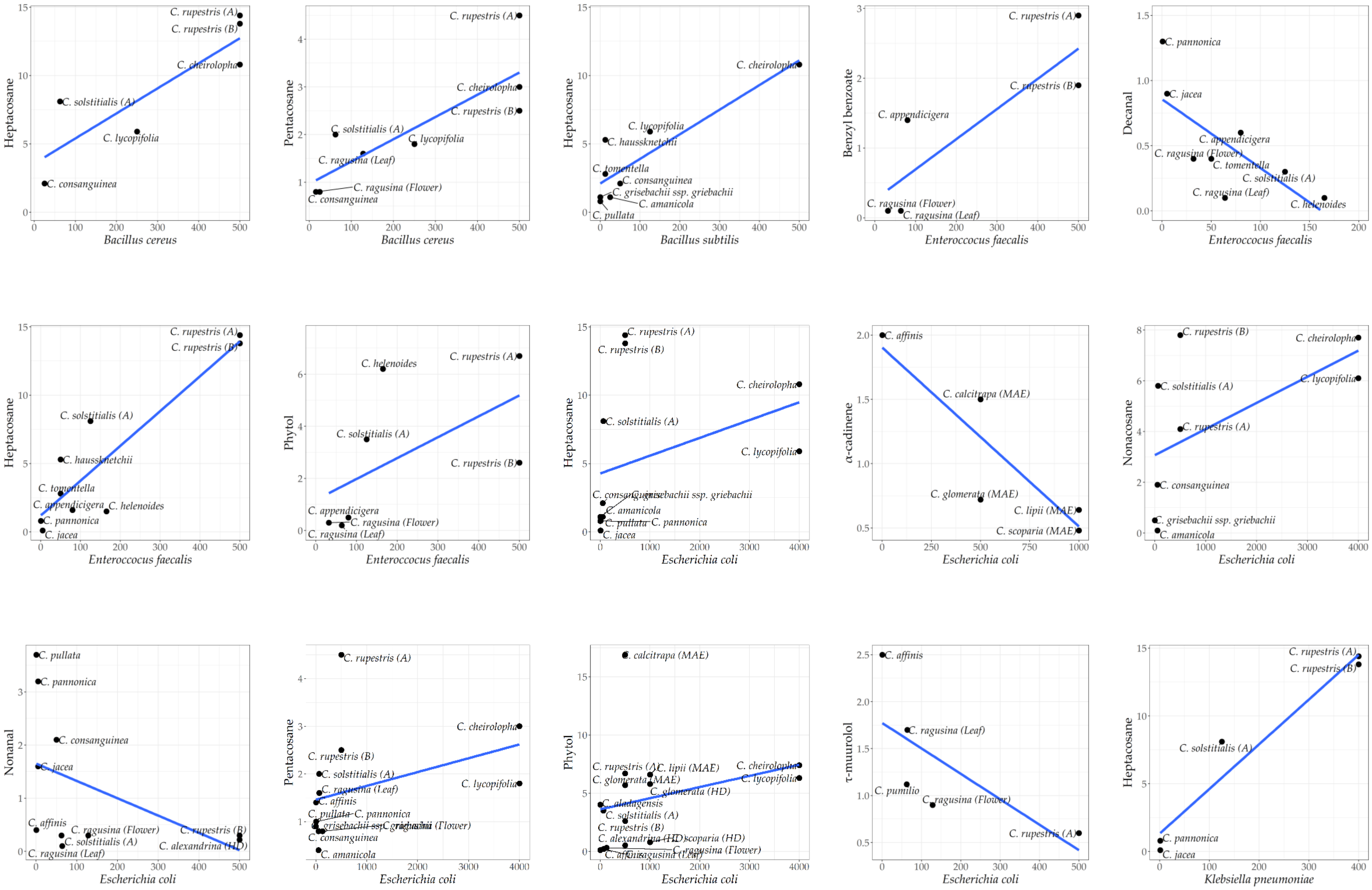

Heptacosane
Bacillus cereus
C. rupestris (A)
C. rupestris (B)
C. cheirolopha
C. solstitialis (A)
C. lycopifolia
C. consanguinea
Pentacosane
Bacillus cereus
C. rupestris (A)
C. cheirolopha
C. rupestris (B)
C. solstitialis (A)
C. lycopifolia
C. ragusina (Leaf)
C. ragusina (Flower)
C. consanguinea
Heptacosane
Bacillus subtilis
C. cheirolopha
C. lycopifolia
C. haussknetchii
C. tomentella
C. consanguinea
C. grisebachii ssp. griebachii
C. amanicola
C. pullata
Benzyl benzoate
Enteroccocus faecalis
C. rupestris (A)
C. rupestris (B)
C. appendicigera
C. ragusina (Flower)
C. ragusina (Leaf)
Decanal
Enteroccocus faecalis
C. pannonica
C. jacea
C. appendicigera
C. ragusina (Flower)
C. tomentella
C. solstitialis (A)
C. ragusina (Leaf)
C. helenoides
Heptacosane
Enteroccocus faecalis
C. rupestris (A)
C. rupestris (B)
C. solstitialis (A)
C. haussknetchii
C. tomentella
C. appendicigera
C. helenoides
C. pannonica
C. jacea
Phytol
Enteroccocus faecalis
C. helenoides
C. rupestris (A)
C. solstitialis (A)
C. rupestris (B)
C. appendicigera
C. ragusina (Flower)
C. ragusina (Leaf)
Heptacosane
Escherichia coli
C. rupestris (A)
C. rupestris (B)
C. cheirolopha
C. solstitialis (A)
C. lycopifolia
C. consanguinea
C. grisebachii ssp. griebachii
C. amanicola
C. pullata
C. pannonica
C. jacea
α-cadinene
Escherichia coli
C. affinis
C. calcitrapa (MAE)
C. glomerata (MAE)
C. lipii (MAE)
C. scoparia (MAE)
Nonacosane
Escherichia coli
C. rupestris (B)
C. cheirolopha
C. solstitialis (A)
C. lycopifolia
C. rupestris (A)
C. consanguinea
C. grisebachii ssp. griebachii
C. amanicola
Nonanal
Escherichia coli
C. pullata
C. pannonica
C. consanguinea
C. jacea
C. affinis
C. ragusina (Flower)
C. rupestris (B)
C. solstitialis (A)
C. alexandrina (HD)
C. ragusina (Leaf)
Pentacosane
Escherichia coli
C. rupestris (A)
C. cheirolopha
C. rupestris (B)
C. solstitialis (A)
C. lycopifolia
C. ragusina (Leaf)
C. affinis
C. pullata
C. pannonica
C. grisebachii ssp. griebachii
C. ragusina (Flower)
C. consanguinea
C. amanicola
Phytol
Escherichia coli
C. calcitrapa (MAE)
C. rupestris (A)
C. lipii (MAE)
C. cheirolopha
C. glomerata (MAE)
C. lycopifolia
C. glomerata (HD)
C. aladagensis
C. solstitialis (A)
C. rupestris (B)
C. alexandrina (HD)
C. scoparia (HD)
C. ragusina (Flower)
C. affinis
C. ragusina (Leaf)
τ-muurolol
Escherichia coli
C. affinis
C. ragusina (Leaf)
C. pumilio
C. ragusina (Flower)
C. rupestris (A)
Heptacosane
Klebsiella pneumoniae
C. rupestris (A)
C. rupestris (B)
C. solstitialis (A)
C. pannonica
C. jacea

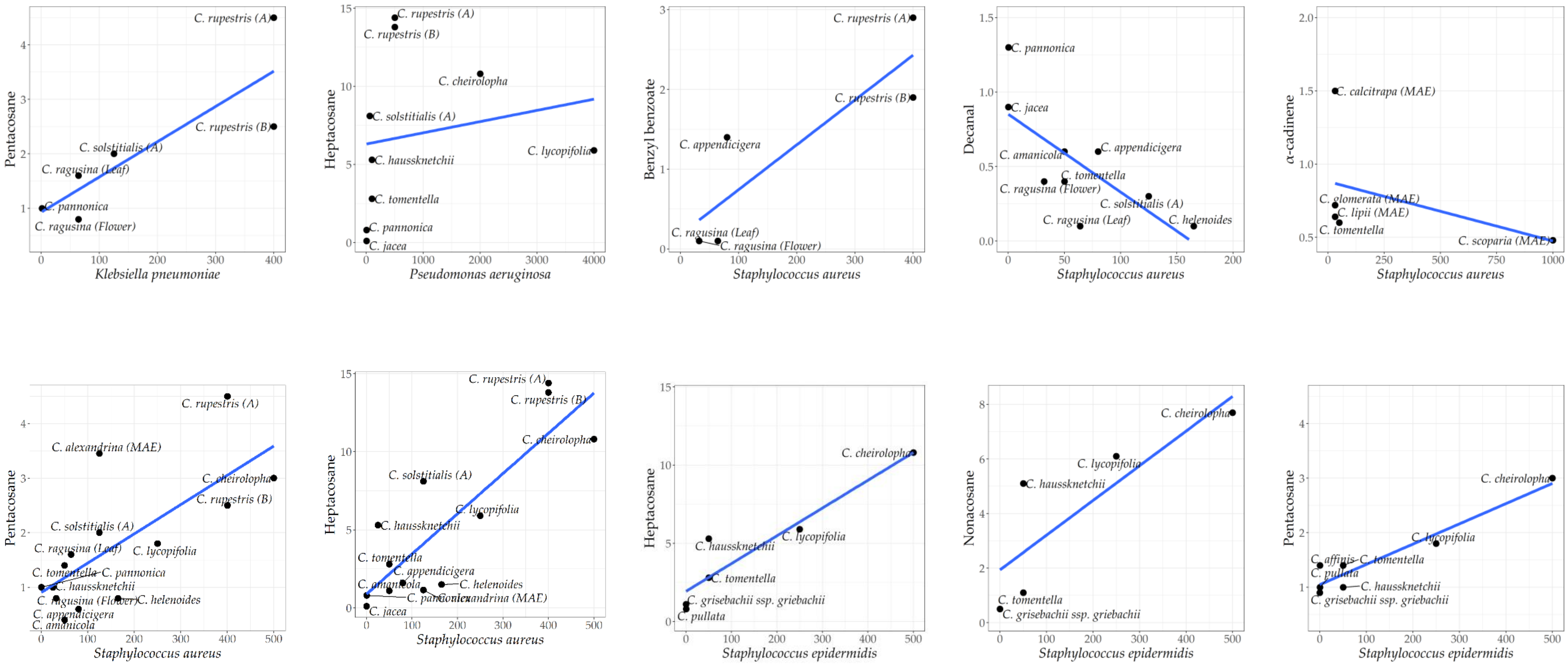

Figure S1. Individual scatterplots showing the statistically significant correlation between specific chemical compound and antimicrobial activity on specific pathogen for various Centaurea essential oils
